# A Nomogram Model to Predict Deep Vein Thrombosis Risk After Surgery in Patients with Hip Fractures

**DOI:** 10.1007/s43465-023-01074-3

**Published:** 2024-01-05

**Authors:** Ruting Bo, Xiaoyu Chen, Xiuwei Zheng, Yang Yang, Bing Dai, Yu Yuan

**Affiliations:** 1 Department of Ultrasound, Tianjin Hospital, Tianjin Hexi District Jiefangnan Road, Tianjin, 300211 China; 2 Clinical Medical College of Tianjin Medical University, Tianjin, 300276 China; 3 Department of Hip Surgery, Tianjin Hospital, Tianjin, 300211 China; 4 Department of Vascular Surgery, Tianjin Hospital, Tianjin, 300211 China

**Keywords:** Hip fracture, Deep vein thrombosis, Nomogram model, Risk factors

## Abstract

**Aims:**

This study aimed to establish a nomogram model for predicting the probability of postoperative deep vein thrombosis (DVT) risk in patients with hip fractures.

**Methods:**

504 patients were randomly assigned to the training set and validation set, and then divided into a DVT group and a non-DVT group. The study analysed the risk factors for DVT using univariate and multivariate analyses. Based on these parameters, a nomogram model was constructed and validated. The predicting performance of nomogram was evaluated by discrimination, calibration, and clinical usefulness.

**Results:**

The predictors contained in the nomogram model included age, surgical approach, 1-day postoperative *D*-dimer value and admission ultrasound diagnosis of the lower limb vein. Furthermore, the area under the ROC curve (AUC) for the specific DVT risk-stratification nomogram model (0.815; 95% CI 0.746–0.884) was significantly higher than the current model (Caprini) (0.659; 95% CI 0.572–0.746, *P* < 0.05). According to the calibration plots, the prediction and actual observation were in good agreement. In the range of threshold probabilities of 0.2–0.8, the predictive performance of the model on DVT risk could be maximized.

**Conclusions:**

The current predictive model could serve as a reliable tool to quantify the possibility of postoperative DVT in hip fractures patients.

## Introduction

DVT refers to thrombosis of the venous lumen after blood clotting abnormally in deep veins, causing venous reflux disorder, limb swelling, and pain [1]. DVT is a common complication of orthopaedic trauma patients and can lead to chronic pain, secondary varicose veins, ulcers and other serious effects on patients' quality of life [2]. Distal venous thrombosis without effective anticoagulant treatment could develop into proximal venous thrombosis [3]. The floating venous thrombosis, in particular, needs to be treated in time to prevent it from spreading to the proximal vein or even causing pulmonary embolism. As the most serious complication of DVT, the mortality rate of pulmonary embolism was as high as 20–30% [4]. The clinical symptoms and signs of DVT are easily neglected due to the influence of fracture and soft tissue injury. After the fracture, the blood is in a hypercoagulable state, and the surgery is the main treatment for hip fracture. Trauma, limb fixation, surgery and other factors will increase the risk of DVT, so early preventive measures should be taken [2]. Zhao et al. [5] found that patients with hip fractures should take medication to prevent DVT. Fu et al. [6] concluded that the incidence of preoperative DVT was 32%, and even after receiving anticoagulant therapy, the incidence of postoperative DVT increased to 56%. The high incidence rate and poor clinical results are concerning. Therefore, it is very important to identify the prognostic factors of DVT after hip fracture surgery and select an individualized treatment strategy.

At present, thrombus risk assessment tools, such as Wells [7], Caprini [8], Padua score [9] and Khorana score [10], are employed to assess the risk of thrombosis, but there are controversial results regarding their effectiveness and validity. For example, the Wells score may be limited by its reliance on subjective judgment and poor specificity, rendering it unsuitable for patients with acute trauma. The Caprini's risk assessment model, another commonly utilized tool, appears excessively unwieldy and intricate to facilitate prompt clinical diagnosis. The Padua score and Khorana score are more appropriate for identifying cancer patients at higher risk for DVT.

A nomogram is a method of visualizing complex mathematical models that consider multiple risk factors, predict disease prognoses, and displaying them in an intuitive manner for clinical treatment guidance [11–13]. At present, the nomograph model is widely used, such as in the prognosis research and risk assessment of cancer patients [14–16], and in predicting the risk of postoperative VTE of malignant tumors [17–20]. However, reports on the use of a nomogram model for predicting the risk of thrombosis after fractures were limited [21]. Despite the prevalence of research on thrombosis risk factors, there remains a stark lack of accurate predictive models for postoperative DVT in hip fracture patients. In response, we attempted to establish a nomogram model to predict the risk of postoperative DVT in patients with hip fractures.

## Materials and Methods

### Patients

We retrospectively analysed 504 patients with hip fracture treated in our hospital between October 2020 and August 2021. This paper was approved by the Hospital's Medical Ethics Committee with the ethical approval number 2022–080. Due to retrospective design of the study the informed consent was waived. The inclusion criteria of this study were as follows: (a) the first hip fracture surgery, (b) time from fracture to surgery ≤ 2 weeks, (c) age ≥ 16, and (d) no previous history of thromboembolism. The exclusion criteria included the following: (1) patients with previous haematological diseases, (2) malignant tumour, abnormal liver and kidney function, (3) patients receiving anticoagulant therapy prior fractures, (4) preoperatively diagnosed patients with DVT, and (5) incomplete data.

### Diagnosis of Thrombus

Vascular ultrasound examination was used in this study to diagnose DVT, with DVT occurring within 3 days of surgery considered as the outcome variable. Patients included in this study were categorized into a DVT group and a non-DVT group based on the occurrence of the outcome variable.

Diagnostic criteria for venous thrombosis [22, 23]: (a) the venous lumen was hypoechoic or anechoic, (b) the venous lumen did not collapse after venous compression (pressure should only be applied if there is no floating thrombus), c. no colour blood flow signal or defect of blood flow filling in the venous lumen, and d. no spectrum signal in the venous lumen.

### Antithrombotic Treatment

According to the guidelines of antithrombotic therapy for DVT [24], after admission, all patients without anticoagulant contraindications received subcutaneous abdominal injection of nadroparin calcium (GlaxoSmithKline, England, 0.4 ml, once a day) combined with a plantar vein pump (30 min, 2 times per day), and anticoagulants were discontinued 24 h before surgery. Prophylactic anticoagulation should be given 24 h after surgery even if the patient did not develop DVT, and the patients in the thrombus group stopped using the plantar vein pump and were injected with nadroparin calcium (2 times per day). If the thrombus was located in the patient's popliteal vein or its proximal segmental vein, interventional therapy was needed, and an inferior vena cava filter was placed, followed by anticoagulant therapy. Patients were required to take rivaroxaban orally (20 mg, once a day) from discharge to postoperative Day 35.

### Clinicopathological Properties

**Fig. 1:**
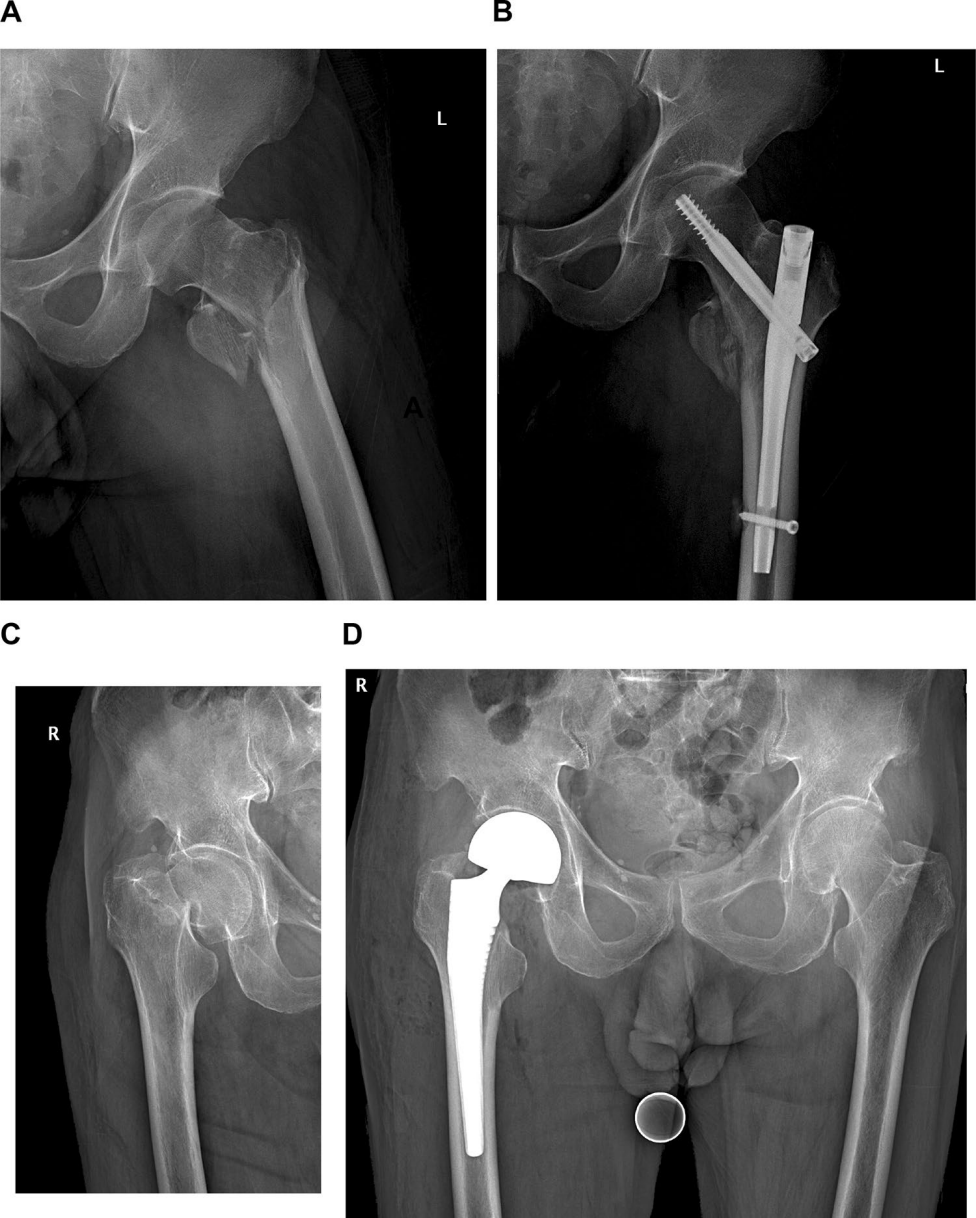
The radiographs of two major ways of fracture fixation in cases. X-ray images of a patient with intertrochanteric fracture before (**A**) and after RIF (**B**). X-ray images of a patient with femoral neck fracture before (**C**) and after HR (**D**)

In this study, potential factors affecting thrombosis were used as predictors through a literature search and clinical judgement. The clinicopathological variables assessed were age, sex, hypertension, diabetes, cerebrovascular disease, cardiovascular disease, fracture side, fracture type, ASA, TO, surgical approach, TIO, PLT, *D*-*d* A, *D*-*d* P, Fib, and admission US. Fracture types included intertrochanteric (Type I), femoral neck (Type II) and hip fractures combined with other fractures (Type III). The surgical approach included HR and RIF. The radiographs of two major ways of fracture fixation in cases were shown in Fig. 1. *D*-dimer was determined by a *D*-dimer detection kit (Qpad, Shanghai Opp Biomedical Co., LTD, China), and a postoperative *D*-dimer level ≥ 450 ng/mL was considered positive.

### Statistical Analysis

Continuous data were compared using independent sample *t* tests when expressed as the mean and standard deviation (SD) and Mann–Whitney *U* tests when expressed as the median. When all categorical variables were expressed in numbers and proportions, the Pearson chi square test was used to compare the differences in these variables between patients. Univariate analysis was regarded as a screening method to determine meaningful variables (*P* < 0.05) for multivariate analysis. According to the multivariate analysis results of binary logistic regression, the prediction model was established and further optimized by stepwise backwards selection. SPSS software (Version 25, SPSS Inc., IBM, NY, USA) was used for univariate, multivariate and binary logistic regression analyses.

According to the results of multivariate analysis, a nomogram was constructed to predict postoperative DVT. The RMS software package in R software (Version 4.1.2, R Foundation for Statistical Computing) was used to build the nomogram model. Internal verification was conducted with the bootstrap method. After bias correction, we compared, predicted and observed outcomes to construct a calibration curve. Evaluations of the accuracy of the model and the net clinical benefit were made using the AUC and DCA. Further, the comparison of nomogram model with current model (Caprini) was performed by comparing ROC curves using the DeLong’s test. The difference was statistically at *P* < 0.05.

## Results

### Baseline Characteristics of All Patients

A total of 504 patients were randomly allocated to the training and validation sets. The two samples basically maintained the similarity between each index (Table 1). The distribution of operation time was different between the two groups, but the median and confidence interval were consistent. The proportion of postoperative *D*-dimer (< 450 ng/ml) in the training set was lower than that in the validation set (41.1% vs. 55.6%), while the proportion of postoperative *D*-dimer (≥ 450 ng/ml) in the training set was higher than that in the verification set (58.9% vs. 44.4%).

**Table 1:** Patient characteristics of risk factors associated with thrombosis

Variables	All patients (*n* = 504)	Training set (*n* = 353)	Validation set (*n* = 151)	*P* value
Sex				0.624
Female	319 (63.3%)	221 (62.6%)	98 (64.9%)	
Male	185 (36.7%)	132 (37.4%)	53 (35.1%)	
Age (years)				0.402
< 70	238 (47.2%)	171 (48.4%)	67 (44.4%)	
≥ 70	266 (52.8%)	182 (51.6%)	84 (55.6%)	
Hypertension				0.484
No	255 (50.6%)	175 (49.6%)	80 (53.0%)	
Yes	249 (49.4%)	178 (50.4%)	71 (47.0%)	
Diabetes				0.046
No	383 (76.0%)	277 (78.5%)	106 (70.2%)	
Yes	121 (24.0%)	76 (21.5%)	45 (29.8%)	
Cerebrovascular diseases				0.202
No	421 (83.5%)	290 (82.2%)	131 (86.8%)	
Yes	83 (16.5%)	63 (17.8%)	20 (13.2%)	
Cardiovascular disease				0.202
No	391 (77.6%)	279 (79.0%)	112 (74.2%)	
Yes	113 (22.4%)	74 (21.0%)	39 (25.8%)	
Medical diseases				0.040
< 3	452 (89.7%)	323 (91.5%)	129 (85.4%)	
≥ 3	52 (10.3%)	30 (8.5%)	22 (14.6%)	
Fracture side				0.272
Left side	289 (57.3%)	208 (58.9%)	81 (53.6%)	
Right side	215 (42.7%)	145 (41.1%)	70 (46.4%)	
Fracture type				0.017
Type I	182 (36.1%)	114 (32.3%)	68 (45.0%)	
Type II	278 (55.2%)	204 (57.8%)	74 (49.0%)	
Type III	44 (8.7%)	35 (9.9%)	9 (6.0%)	
ASA				0.821
I	43 (8.5%)	29 (8.2%)	14 (9.3%)	
II	147 (29.2%)	105 (29.7%)	42 (27.8%)	
III	310 (61.5%)	217 (61.5%)	93 (61.6%)	
IV	4 (0.8%)	2 (0.6%)	2 (1.3%)	
TO (h)	2 (1–3)	2 (1–3)	2 (1–3)	0.007
Surgical approach				0.002
HR	236 (46.8%)	181 (51.3%)	55 (36.4%)	
RIF	268 (53.2%)	172 (48.7%)	96 (63.6%)	
TIO (h)	61 (14–384)	61 (14–384)	61 (16–364)	0.703
PLT (× 10^9^/L)	197 (65–583)	198 (65–470)	196 (83–583)	0.961
*D*-*d* *A* (ng/ml)	1400 (91–16,000)	1500 (100–16000)	1200 (91–16,000)	0.018
*D*-*d* *P* (ng/ml)				0.003
< 450	229 (45.4%)	145 (41.1%)	84 (55.6%)	
≥ 450	275 (54.6%)	208 (58.9%)	67 (44.4%)	
Fib (g/L)	3.88 (1.65–7.21)	3.83 (1.65–7.21)	3.93 (2.25–6.53)	0.220
Admission US				0.163
Non-DVT	403 (77.3%)	288 (81.6%)	115 (76.2%)	
DVT	101 (22.7%)	65 (18.4%)	36 (23.8%)	

In our research, there were 221 cases in the non-DVT set and 132 cases in the DVT group in the training set (Table 2). Table 2 presented a comparison of the patient’s clinical characteristics between the DVT group and the non-DVT group. There was no statistically significant difference between the two groups in hypertension, diabetes, cerebrovascular disease, cardiovascular disease, fracture side, ASA score, TO, TIO, PLT, Fib or admission *D*-dimer.

**Table 2:** Univariate analysis of factors associated with post-surgical DVT in hip fractures

Variables	Non-DVT (*n* = 221)	DVT (*n* = 132)	*P* value
Sex			0.592
Female	136 (61.5%)	85 (64.4%)	
Male	85 (38.5%)	47 (35.6%)	
Age (years)			
< 70	126 (57.0%)	45 (34.1%)	0.000
≥ 70	95 (43.0%)	87 (65.9%)	
Hypertension			0.573
No	107 (48.4%)	68 (51.5%)	
Yes	114 (51.6%)	64 (48.5%)	
Diabetes			0.704
No	172 (77.8%)	105 (79.5%)	
Yes	49 (22.2%)	27 (20.5%)	
Cerebrovascular diseases			0.873
No	181 (81.9%)	109 (82.6%)	
Yes	40 (18.1%)	23 (17.4%)	
Cardiovascular disease			
No	175 (79.2%)	104 (78.8%)	0.929
Yes	46 (20.8%)	28 (21.2%)	
Medical diseases			0.382
< 3	200 (90.5%)	123 (93.2%)	
≥ 3	21 (9.5%)	9 (6.8%)	
Fracture side			
Left side	123 (55.7%)	85 (64.4%)	0.106
Right side	98 (44.3%)	47 (35.6%)	
Fracture type			
Type I	59 (26.7%)	55 (41.7%)	0.012
Type II	140 (63.3%)	64 (48.5%)	
Type III	22 (10.0%)	13 (9.8%)	
ASA			
I	20 (9%)	9 (6.8%)	0.562
II	70 (31.7%)	35 (26.5%)	
III	130 (58.8%)	87 (65.9%)	
IV	1 (0.5%)	1 (0.8%)	
TO (h)	1.68 ± 0.622	1.76 ± 0.628	0.506
Surgical approach			0.033
HR	123 (55.7%)	58 (43.9%)	
RIF	98 (44.3%)	74 (56.1%)	
TIO (h)	58 (14–384)	63 (16–336)	0.196
PLT (× 10^9^/L)	199 (65–470)	197 (68–462)	0.729
*D*-*d* *A* (ng/ml)	1500 (100–15000)	1500 (100–16000)	0.725
*D*-*d* *P* (ng/ml)			
< 450	111 (50.2%)	34 (25.8%)	0.000
≥ 450	110 (49.8%)	98 (74.2%)	
Fib (g/L)	3.83 (2.10–7.21)	3.70 (1.65–6.72)	0.409
Admission US			0.000
Non-DVT	213 (96.4%)	75 (56.8%)	
DVT	8 (3.6%)	57 (43.2%)	

### Construction of the Nomogram

We performed univariate logistic regression analysis in the training set. The variables of age, fracture type, surgical approach, *D*-*d* P, and admission US were found to be significantly correlated with postoperative DVT in hip fractures (all, *P* < 0.05) (Table 2). Multivariate logistic regression analysis was performed on the identified potential predictors of thrombosis, and the results showed that age, surgical approach, *D*-*d* P and admission US were considered to be independent risk factors for postoperative DVT of hip fractures (all, *P* < 0.05) (Table 3).

**Table 3:** Multivariate analysis of factors associated with post-surgical DVT in hip fractures

Variables	*β*	SE	Wald *χ*^2^	*P*	Exp (B)	95% CI
Age	0.590	0.269	4.798	0.028	1.803	1.064–3.056
Surgical approach	0.731	0.278	6.906	0.009	2.078	1.204–3.585
*D*-*d* *P* (ng/ml)	1.321	0.302	19.083	0.000	3.784	2.072–6.780
Admission US	2.937	0.421	48.586	0.000	18.855	8.257–43.059

**Fig. 2:**
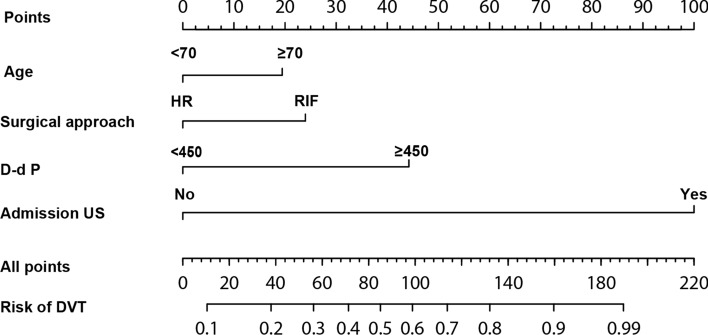
Nomogram predicting the postoperative DVT in hip fractures

**Fig. 3:**
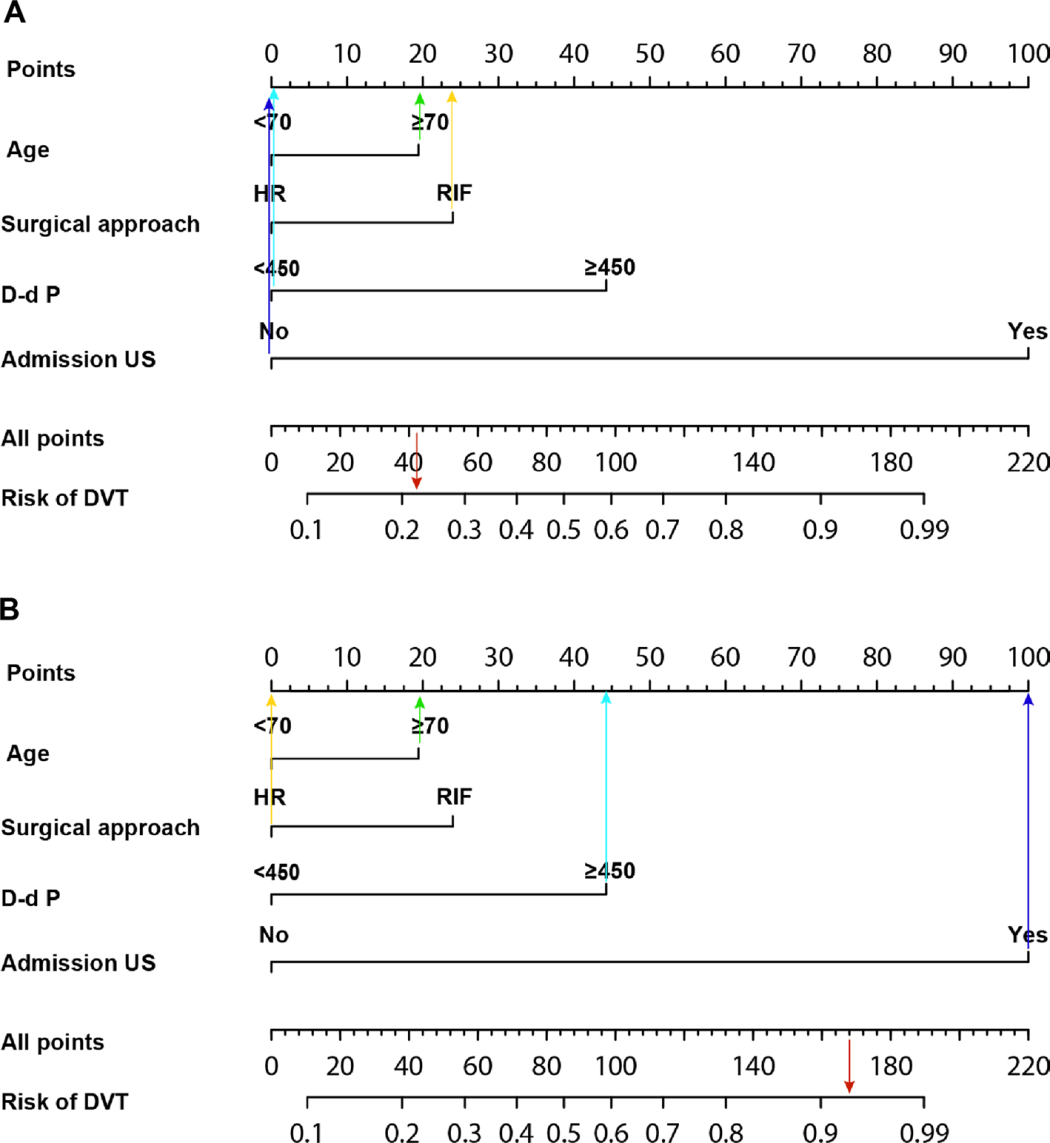
Examples of clinical application of the nomogram. **A** The nomogram of patient 1 resulted in a total score of 42 points for age ≥ 70 years (19 points), RIF (23 points), *D*-dimer < 450 ng/ml (0 points), negative on admission US (0 points). The corresponding risk of DVT was 0.42, and the actual result of the patient was negative for DVT after hip fracture surgery. **B** The nomogram of patient 2 resulted in a total score of 165 points for age ≥ 70 years (19 points), HR (0 points), *D*-dimer ≥ 450 ng/ml (47 points), positive on admission US (99 points). The corresponding risk of DVT was 0.93, and the actual result of the patient was positive for DVT after hip fracture surgery

We developed a nomogram model of postoperative DVT for hip fractures based on multivariate analyses in this study for the first time (Fig. 2). We used this nomogram prediction model to calculate the incidence of postoperative DVT. Representative examples of predicting the risk of postoperative DVT in hip fractures patients were shown in Fig. 3. The probability of DVT using the nomogram model was 42% in patient 1, and the actuarial result of the patient was negative for DVT after hip fracture surgery (Fig. 3A). The probability of DVT using the nomogram model was 93% in patient 2, and the actuarial result of the patient was positive for DVT after hip fracture surgery (Fig. 3B). As the nomogram indicated, the higher the patient's score, the greater their risk of developing postoperative DVT.

### Validation and Performance of the Nomogram

**Fig. 4:**
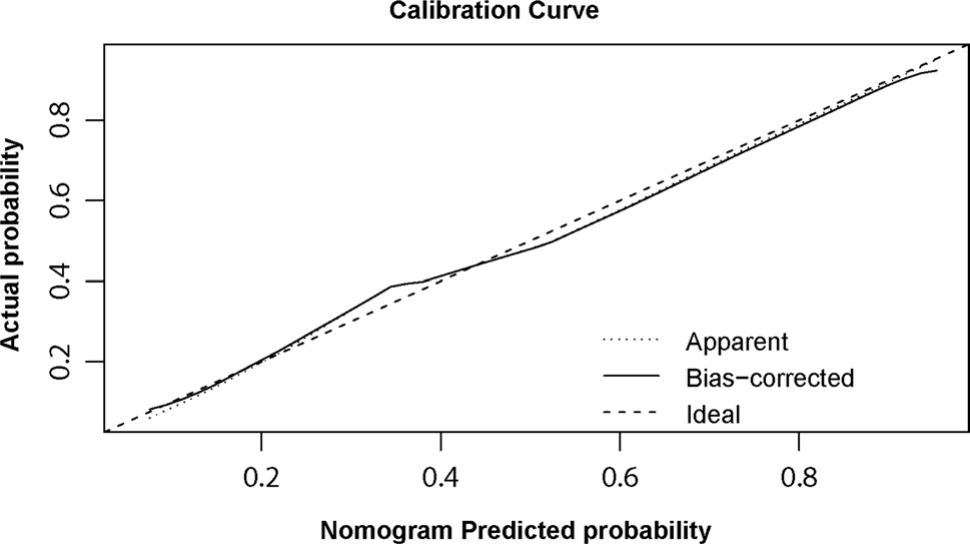
Calibration plot of the nomogram for the probability of postoperative thrombosis in hip fractures (bootstrap 1000 repetitions)

**Fig. 5:**
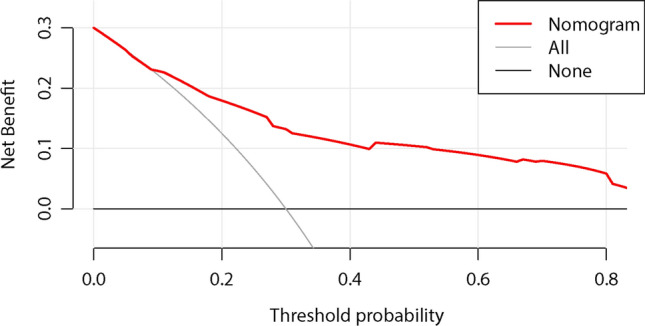
DCA of the nomogram of postoperative DVT of hip fractures. The red line represented the nomogram. The black line (no intervention) represented all negative samples. The grey line (received intervention) represented all positive samples

**Fig. 6:**
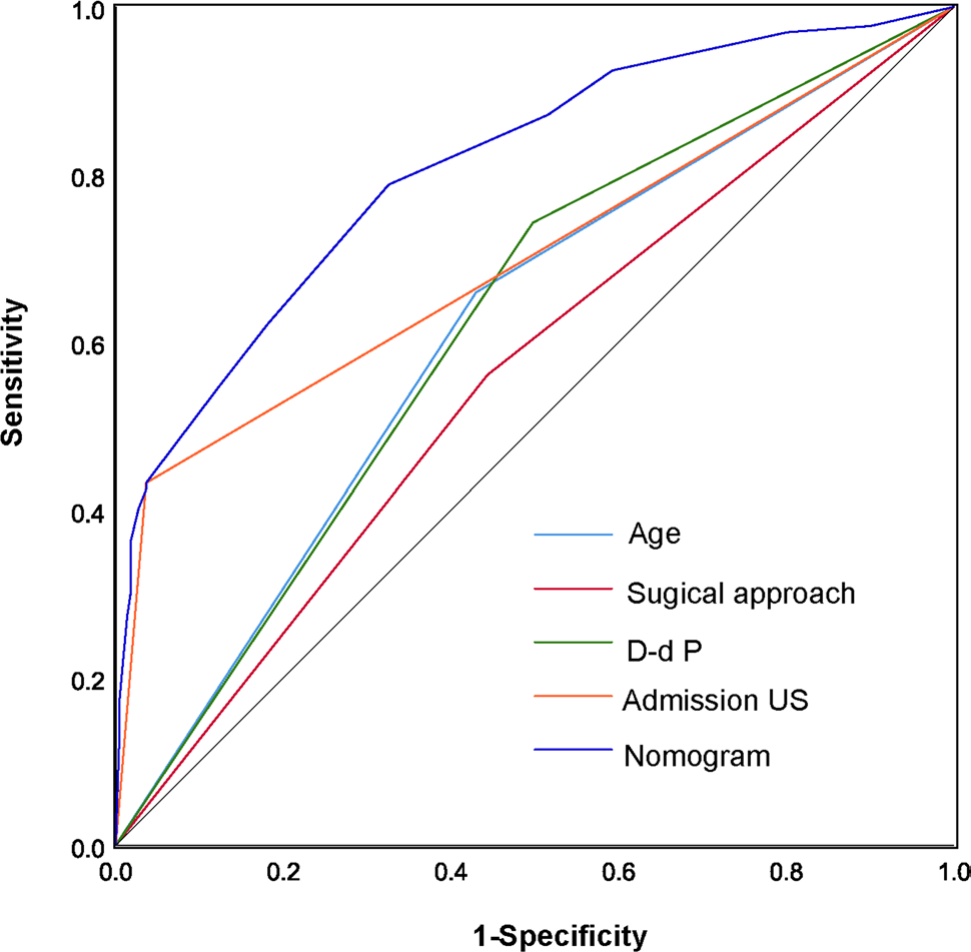
ROC curve analysis of related indicators of postoperative DVT after hip fractures

The consistency was verified by a calibration curve. Figure 4 showed that there were favourable consistencies between the predicted and actual risk of postoperative DVT, indicating that the nomogram had a good correction effect on the predicted probability of postoperative DVT for patients with hip fractures. To quantify the overall net benefits of different threshold probabilities, DCA was performed to determine the clinical utility of the developed nomogram. As shown in Fig. 5, DCA showed that the prediction performance of DVT risk could obtain the maximum benefit in the range of threshold probability of 0.2–0.8. The net benefit reflected the balance between DVT risk and the potential cost of unnecessary prevention of thrombosis. To assess the discrimination of the prediction model, we computed the AUC of the ROC for the training set. As shown in Fig. 6, the AUC of the nomogram was 0.808 (95% CI 0.761–0.856). The AUCs of age, surgical approach, postoperative *D*-dimer and admission US were 0.615 (95% CI 0.554–0.675), 0.559 (95% CI 0.497–0.620), 0.622 (95% CI 0.563–0.682) and 0.698 (95% CI 0.637–0.759), respectively. The results showed that the model possessed a high discriminating power.

### Comparison with the Caprini Score

**Fig. 7:**
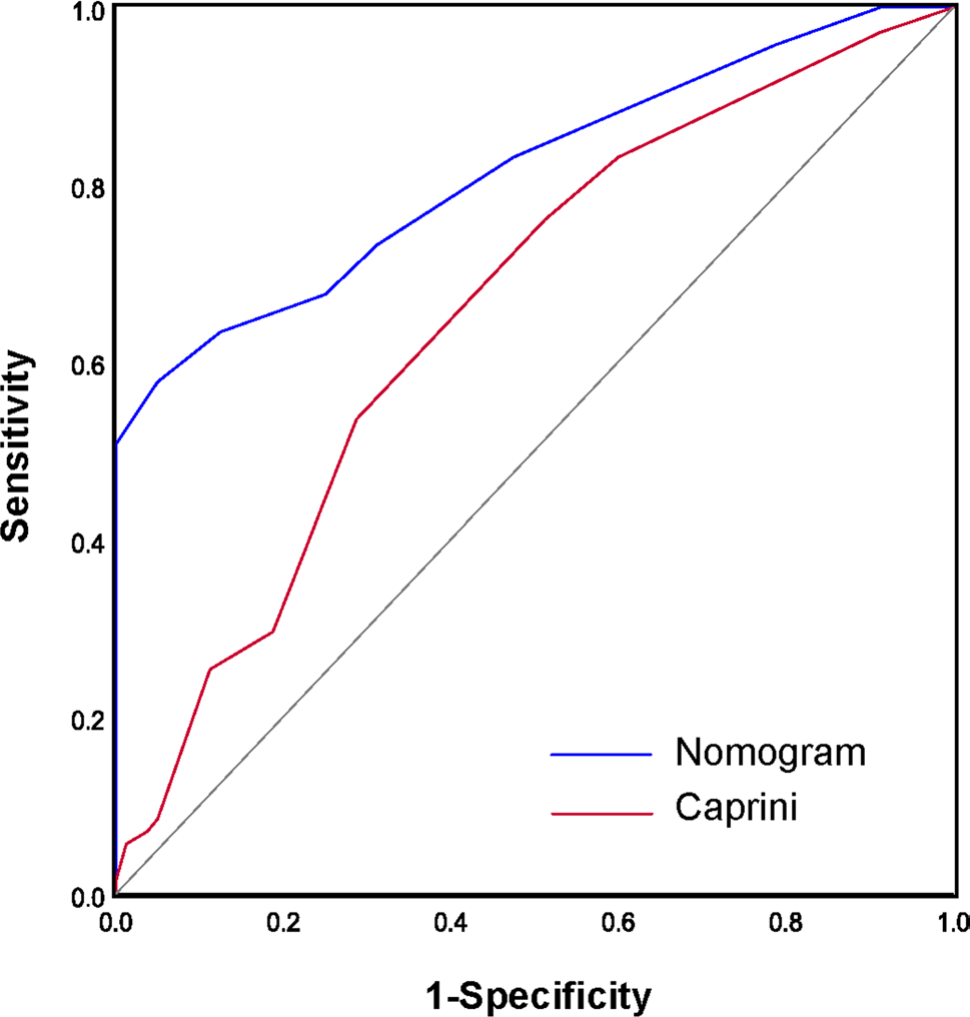
ROC curve analysis of the nomogram model and the Caprini score in validation set (AUC = 0.815 vs. 0.659, *P* < 0.05)

We then validated our nomogram model in the training set and its performance was externally compared with the Caprini. The ROC curves of the two risk assessment models were shown in Fig. 7. The AUC for the nomogram model (0.815; 95% CI 0.746–0.884) was significantly higher than the Caprini (0.659; 95% CI 0.572–0.746). The results demonstrated that the nomogram model had better predictive value (*P* < 0.05).

## Discussion

Zhang et al. concluded that the incidence rate of DVT after hip fracture surgery was 57.23% [25], whereas Song et al. argued that the incidence of DVT after hip fracture surgery was 32.8% [26]. The results of our research showed that the incidence of DVT after hip fracture surgery was 37%. Therefore, we concluded that the incidence of thrombosis with hip fractures was high. Systematic identification of individual susceptibility to DVT could provide an opportunity to reduce the risk of thrombotic complications. At present, studies on the thrombosis risk of patients with hip fractures are based on the analysis of risk factors, and there is a lack of accurate prediction model of individual risk among patient [18]. In this study, 504 hip patients were retrospectively analysed, and a reliable nomogram model was established so that clinicians could predict the likelihood of DVT in the patient and contribute to the prevention and treatment of thrombosis.

In this study, multivariate logistic regression analysis revealed that age ≥ 70 years, *D*-dimer ≥ 450 ng/ml, RIF, and preoperative DVT after admission were independently associated with postoperative DVT. The following was an analysis.

Some researchers believe that age is an independent risk factor for DVT. Our study confirmed that ages ≥ 70 years were independently correlated with postoperative DVT in patients after hip fractures [27, 28]. Shahi et al. [29] and Zhao K et al. [5] found similar results to ours. This may be related to elderly patients having a higher viscosity of whole blood, reduced fibrous protein activity, and worse lower body function in patients with vascular elasticity, the body's stress response after trauma, and aggravated blood coagulation mechanism imbalance. In addition, elderly patients experience slower postoperative recovery, stay in bed longer, have decreased activity, and thus clogged injured limb blood flow, which easily induces the occurrence of DVT [23].

*D*-dimer is a fibrin degradation product that reflects thrombin production and is a sensitive indicator of fibrinolytic system activity [30]. *D*-dimer is affected by a variety of factors, such as trauma, surgery, tumour, infection, disseminated intravascular coagulation, etc. [31], but *D*-dimer is still an important factor for the clinical monitoring of thrombosis [32]. Our study found that postoperative *D*-dimer ≥ 450 ng/mL was one of the indicators for the evaluation of postoperative DVT, and *D*-dimer could be used as one of the reference indicators for the occurrence of postoperative DVT after hip fractures, which was consistent with the results of previous papers [33]. Therefore, the detection of postoperative *D*-dimer was of great significance for the early diagnosis of postoperative DVT in patients with hip fractures.

Some studies suggested that upon admission diagnosis for acute DVT in hip fracture patients, even after treatment with thrombolysis, the incidence of DVT after surgery was still relatively high [6]. In this paper, we found the same results and confirmed that DVT formation in patients with hip fractures at admission had important reference value for postoperative DVT evaluation. Therefore, attention and treatment should be given to patients with DVT formation at admission to prevent further development of thrombosis or the formation of chronic thrombosis, resulting in a series of complications.

In our study, 96.7% of 182 intertrochanteric fractures in type I underwent reduction surgery, and 77% of 278 femoral neck fractures in type II underwent hip replacement surgery. Fracture type was a variable related to development of postoperative DVT in univariate analysis, but it was meaningless in multivariate analysis, because fracture type and surgical approach almost formed a one-to-one correspondence, which created a spurious association between fracture type and thrombosis. In fact, the surgical approach was the independent risk factors for postoperative DVT of hip fractures in our paper. Nowadays, HR has been considered the gold standard for displaced femoral neck fractures [34]. HR is less invasive allows elderly patients to faster mobilize and recovery, as compared to RIF [34]. In orthopaedic surgery, the risk of thrombosis could generally be considered as proportional to the duration and invasiveness (trauma, demolition) of the surgical procedure [35]. A longer operation time and more intraoperative blood loss would increase patients' blood hypercoagulability and significantly stimulate the body's stress response, thereby increasing the risk of thrombosis [36]. Compared with the intra-articular fractures of hip arthroplasty, patients with extra-articular fractures undergoing RIF are often relatively more serious, the surgical procedure is more complicated, and the local blood transport around the extra-articular fractures is rich, which easily causes more blood loss [34]. Therefore, unlike RIF, HR is thought to have a lower risk of complications such as thrombotic events [34], which is consistent with our results.

Our research concluded that the nomogram model had possessed a high discriminating power. The AUC of the training set and the validation set were 0.808 and 0.815, respectively. Furthermore, the model had good calibration effect and clinical utility. We then compared the predictive value of our nomogram with that of Caprini score, the most widely used tool to assess risk of DVT. It is well known that, the Caprini score is cumbersome with 31 variables and poses challenges with inter-rater reliability [37]. An abbreviated Caprini risk assessment model has been developed to stratify patients at risk of DVT [38]. Ten risk factors were included in the abbreviated model (recent major surgery, length of surgery > 2 h, transfusion, restricted mobility > 72 h, central venous catheter, current major surgery, age, history of VTE, hip or leg fracture, and serious trauma). The abbreviated Caprini score had with similar discriminatory ability to the original Caprini score in trauma patients. This analysis to assess the predictive ability of the nomogram and the abbreviated Caprini score in the same study cohort was performed. The result demonstrated that the nomogram (AUC = 0.815; 95% CI 0.746–0.884) had a better predictive value when compared with the abbreviated Caprini score (AUC = 0.659; 95% CI 0.572–0.746). The reason for the differences may have been due to the scale discrimination in the applicable population. Furthermore, the abbreviated Caprini score for postoperative DVT of hip fractures was still cumbersome, time-consuming to complete and the scale appears to be still complicated for clinical diagnosis. We had created the special nomogram model using the four components that were most highly associated with DVT, also removing all subjective variables, which may increase workflow efficiency and reduce inter-rater variability. The evaluation model was feasible and easy to complete and worked better in settings with limited resources and workforce capacity. For patients at high risk of thrombosis evaluated by the nomogram model, during the perioperative period, physicians can provide complementing physical interventions (plantar pump training, application of elastic socks) as well as medication precautions. Patients with low thrombosis risks should walk immediately after the operation to avoid deep vein thrombosis to lessen patient burden and save medical expenses.

## Limitations

There are some limitations in the present study and further research needs to be done in the future. Firstly, it was a retrospective study by a single centre, selection bias was inevitable which may influence the result. Therefore, a prospective study, preferably a multicenter validation, with larger sample size is needed to determine its generalizability and efficiency. Furthermore, the current work neglected some lab variables associated with a hypercoagulable state. Otherwise, since the study did not provide long-term follow-up of patients, the results may be biased. Finally, the model use limited to simple hip fractures, multiple outcomes (e.g., bleeding events, thrombotic disorder) can be considered in future studies.

## Conclusion

In our research, we have established a beneficial nomogram model using four risk factors to predict the risk of DVT in patients with hip fractures, validated this model and determined its high performance. Moreover, the discriminatory capacity of the nomogram model was superior to that of each variable independently and the Caprini score. Based on the application of the proposed model, clinicians will have the ability to determine with greater accuracy which patients are likely to develop thrombosis and provide them with appropriate adequate prevention and treatment measures.

## Data Availability

The datasets used during the current study are available from the corresponding author on reasonable request.

## Abbreviations

DVT: Deep vein thrombosis
VTE: Venous thrombus embolism
ASA: American Society of Anaesthesiologists score
TO: Operation time
TIO: Injury-operative time interval
PLT: Platelet
*D-d**A*: Admission *D*-dimer
*D*-*d**P*: 1-Day postoperative *D*-dimer
Fib: 1-Day postoperative fibrinogen
Admission US: Admission ultrasound diagnosis results of lower limbs venous
HR: Hip replacement
RIF: Reduction and internal fixation
DCA: Decision curve analysis
